# Aboveground impacts of a belowground invader: how invasive earthworms alter aboveground arthropod communities in a northern North American forest

**DOI:** 10.1098/rsbl.2021.0636

**Published:** 2022-03-30

**Authors:** Malte Jochum, Lise Thouvenot, Olga Ferlian, Romy Zeiss, Bernhard Klarner, Ulrich Pruschitzki, Edward A. Johnson, Nico Eisenhauer

**Affiliations:** ^1^ German Centre for Integrative Biodiversity Research (iDiv) Halle-Jena-Leipzig, Puschstrasse 4, 04103 Leipzig, Germany; ^2^ Leipzig University, Institute of Biology, Puschstrasse 4, 04103 Leipzig, Germany; ^3^ J.F. Blumenbach Institute of Zoology and Anthropology, University of Goettingen, Untere Karspuele 2, Goettingen 37073, Germany; ^4^ Department Biological Sciences, University of Calgary, Calgary, Alberta, Canada T2N 1N4

**Keywords:** belowground, aboveground, earthworm invasion, insect decline, forest, Canada

## Abstract

Declining arthropod communities have recently gained a lot of attention, with climate and land-use change among the most frequently discussed drivers. Here, we focus on a seemingly underrepresented driver of arthropod community decline: biological invasions. For approximately 12 000 years, earthworms have been absent from wide parts of northern North America, but they have been re-introduced with dramatic consequences. Most studies investigating earthworm-invasion impacts focus on the belowground world, resulting in limited knowledge on aboveground-community changes. We present observational data on earthworm, plant and aboveground arthropod communities in 60 plots, distributed across areas with increasing invasion status (low, medium and high) in a Canadian forest. We analysed how earthworm-invasion status and biomass impact aboveground arthropod community abundance, biomass and species richness, and how earthworm impacts cascade across trophic levels. We sampled approximately 13 000 arthropods, dominated by Hemiptera, Diptera, Araneae, Thysanoptera and Hymenoptera. Total arthropod abundance, biomass and species richness declined significantly from areas of low to those with high invasion status, with reductions of 61, 27 and 18%, respectively. Structural equation models suggest that earthworms directly and indirectly impact arthropods across trophic levels. We show that earthworm invasion can alter aboveground multi-trophic arthropod communities and suggest that belowground invasions might be underappreciated drivers of aboveground arthropod decline.

## 1. Introduction

Recent reports on arthropod species richness, abundance and biomass declines [1–3] have triggered concern about ‘the little things that run our world’ [4] and the consequences of their loss. Even though the situation might not be equally bad for all taxa and ecosystem types [5], the extent of the reported negative trends, together with the lack of sufficient long-term datasets to establish such trends across all taxa and ecosystems [6–8], are worrying. With arthropods contributing to central ecosystem processes and services [9], their loss will have unprecedented consequences for ecosystems and human societies.

In order to halt or reverse arthropod decline, we need to understand the underlying drivers. Given their importance as broad global change drivers [10], it is unsurprising that climate and land-use change are prominent examples [1,5,11,12]. However, though underrepresented in research on arthropod declines, other drivers might still play an important role. Here, we focus on one potentially underappreciated driver of arthropod decline: the invasion of a belowground ecosystem engineer, earthworms [13].

Although commonly perceived as having mostly positive impacts on their environment [14,15], earthworms can transform invaded ecosystems [16] that are not able to deal with their impacts on the ecosystems' physical, chemical and biological properties [17–20]. Earthworm invasion is a globally occurring problem [21]. One region with both particularly severe impacts and a lot of research on the consequences is northern North America. Here, most earthworm species present today have been absent since the last glaciation (maximum approximately 20 000, end of cover approximately 12 000 years ago) and have only been re-introduced a few hundred years ago [17,22].

Earthworm invasion alters soil abiotic conditions [17,19], plant communities [23–25] and soil fauna [26–29]. Moreover, there are reports of consequences for aboveground vertebrates, such as salamanders, birds and deer [18,30]. There also are some aboveground invertebrate studies, but these mostly focus on litter-dwelling fauna [28,31]. With invasive earthworms impacting soil abiotic conditions, soil fauna, plants and litter-dwelling arthropods, the open question is whether and how their invasion impacts aboveground, vegetation-dwelling arthropods, and if these changes cascade across trophic levels. For example, earthworms could directly serve aboveground arthropods as a food resource [32] or indirectly affect them via altered habitat structure, resource availability (leaf litter) or plant communities [25,33]. We used observational data on earthworm, plant and aboveground arthropod communities from a Canadian forest to investigate (i) whether belowground invasion by earthworms changes aboveground arthropod communities and, using structural equation models (SEMs), (ii) how earthworms directly and indirectly impact higher trophic levels mediated by plants, herbivores and detritivores. We expected invasive earthworms to decrease the abundance, biomass and diversity of aboveground arthropod communities via cascading effects across trophic levels [18,34].

## 2. Material and methods

We studied a south-facing forest slope above the Northwestern shore of Barrier Lake, Kananaskis Valley, Alberta, Canada (51°02′6″ N, 115°03′54″ W, approximately 1450 m.a.s.l.). The forest is dominated by trembling aspen (*Populus tremuloides*) interspersed with balsam poplar (*Populus balsamifera*), with a dense understorey vegetation and a grey luvisol soil. It has a long history of earthworm-invasion research, including investigations on soil abiotic (soil chemistry and physics) and biotic (micro, meso and macrofauna) aspects [29,30,35–37]. Land-use intensity is low and homogeneous across invasion status areas and the forest last burned in 1909 [29]. We combine community data on earthworms, plants and aboveground arthropods sampled in June and July 2019 on observational plots of the ‘EcoWorm’ project (described in Eisenhauer *et al*. [30]). After verifying earthworm-invasion status along the slope, we established 20 plots of 1 m x 2 m in each of three invasion status areas: low, mid and high invasion (*n* = 60 plots, electronic supplementary material, SuppInfo §S1 and figure S1). These categories differed significantly in earthworm abundance, biomass, species richness and functional group richness (electronic supplementary material, SuppInfo §1, figures S2 and S3). Thus, we focused on invasion status as the main predictor and show responses to earthworm biomass in the electronic supplementary material, SuppInfo. We used 1 m^2^ for plant community assessments and the other half plot for arthropod (1 m^2^) and earthworm sampling (0.25 m^2^; electronic supplementary material, SuppInfo, figure S4). We identified every plant species and estimated total plant cover using a modified decimal scale [38], and we estimated plot-level canopy openness (for details, see electronic supplementary material, SuppInfo §1, figure S5).

Earthworms were extracted using a combination of hand sorting and mustard extraction. Individuals were identified to species level, assigned to a functional group, and their fresh mass was assessed (electronic supplementary material, SuppInfo §S1). We sampled aboveground arthropods using a vacuum suction sampler. All collected animals were hand-sorted, identified to (morpho-)species, assigned to a trophic feeding guild (see electronic supplementary material, SuppInfo §S2 for details, figures S6 and S7, and table S1), and their fresh biomass was estimated (electronic supplementary material, SuppInfo §S3; [39–41]. We calculated abundance, biomass and species richness of all arthropods, and, separately, for herbivores, omnivores (combining all mixed-diet feeding guilds), predators, detritivores and parasitoids. While abundance and biomass were calculated based on all individuals (excluding mites and springtails), species richness was calculated based on adults only.

Data analysis was done in R v. 3.6.3 [42]. We assessed arthropod community responses to invasion using earthworm-invasion status (categorical: low, mid and high) and biomass (continuous, log_10_-transformed) as predictors in separate models for each predictor-response variable combination. For details on these analyses, please see electronic supplementary material, SuppInfo §4. We used the R lavaan 0.6-9 [43] package to construct SEMs testing direct and indirect effects of earthworm invasion on aboveground arthropod abundance, biomass and richness, separately (see electronic supplementary material, SuppInfo §6).

## 3. Results

**Figure 1.**
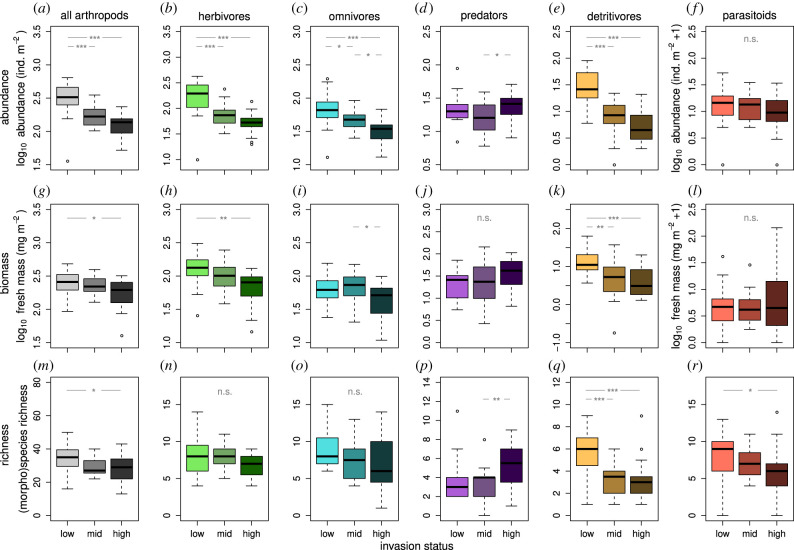
Effects of earthworm-invasion status (low, mid, high; lighter to darker colour shades) on the abundance (*a*–*f*), biomass (*g*–*l*) and (morpho)species richness (*m*–*r*) of total aboveground arthropods (grey), herbivores (green), omnivores (turquoise), predators (purple), detritivores (brown) and parasitoids (red). Asterisks and ‘n.s.’ illustrate significance levels for differences between invasion status categories (‘n.s.’ not significant, ***p ≤ 0.001; **p ≤ 0.01; *p ≤ 0.05; *p* > 0.05). *p*-values are from simple linear models and GLMs with Poisson-distributed response variables (richness models), respectively. *N* = 60. For model results, see table 1.

We collected 13 037 aboveground invertebrates (230 Pulmonata individuals included; for brevity, hereafter: arthropods), 4814 of which were adults (for R-code and data, please see [44]). For taxonomic and trophic details, see electronic supplementary material, SuppInfo figures S6 and S7, and table S1. Arthropod communities differed between invasion status categories (figure 1) and along the observational earthworm biomass gradient (electronic supplementary material, SuppInfo, figure S8). Out of 18 models testing arthropod responses to earthworm-invasion status, 11 found significant negative relationships, while two relationships were positive (figure 1 and table 1). All three total arthropod properties responded negatively to earthworm invasion (at least from ‘low’ to ‘high’ invasion). Predator abundance and richness increased with earthworm-invasion status (mid to high). Out of 18 models testing arthropod responses to increasing earthworm biomass, there were seven significant negative relationships and one significant positive relationship (electronic supplementary material, SuppInfo, figure S8 and table S2). Notably, total arthropod abundance declined, as well as herbivore abundance and biomass, omnivore abundance and detritivore abundance, biomass and richness; only predator biomass increased significantly.

**Table 1.** Results of models relating aboveground arthropod abundance, biomass and (morpho)species richness to invasion status (figure 1). For each model, the table shows the response variable, arthropod group, sample size (*n*), model type, response transformation and *p*-values for Tukey *post hoc* and general linear hypotheses tests (see §2 and electronic supplementary material, SuppInfo parapraph 4). *p*-values significant to an alpha level of 0.05 are italicized. Values are rounded.

response	group	*n*	model type	resp. transf.	*p* low-high	*p* low-mid	*p* mid-high
abundance	all	60	aov	log_10_	*<0.001*	*<0.001*	0.184
abundance	herbivores	60	aov	log_10_	*<0.001*	*<0.001*	0.137
abundance	omnivores	60	aov	log_10_	*<0.001*	*0.040*	*0.030*
abundance	predators	60	aov	log_10_	0.682	0.238	*0.043*
abundance	detritivores	60	aov	log_10_	*<0.001*	*<0.001*	0.424
abundance	parasitoids	60	aov	log_10_(+1)	0.405	0.991	0.480
biomass	all	60	aov	log_10_	*0.042*	0.800	0.166
biomass	herbivores	60	aov	log_10_	*0.002*	0.295	0.113
biomass	omnivores	60	aov	log_10_	0.060	0.845	*0.015*
biomass	predators	60	aov	log_10_	0.135	0.988	0.179
biomass	detritivores	60	aov	log_10_	*<0.001*	*0.002*	0.894
biomass	parasitoids	60	aov	log_10_(+1)	0.859	0.981	0.758
richness	all	60	glm.nb	none	*0.025*	0.058	0.942
richness	herbivores	60	glm	none	0.405	0.998	0.438
richness	omnivores	60	glm	none	0.074	0.199	0.884
richness	predators	60	glm	none	0.067	0.675	*0.007*
richness	detritivores	60	glm	none	*<0.001*	*<0.001*	0.963
richness	parasitoids	60	glm	none	*0.033*	0.519	0.329

**Figure 2.**
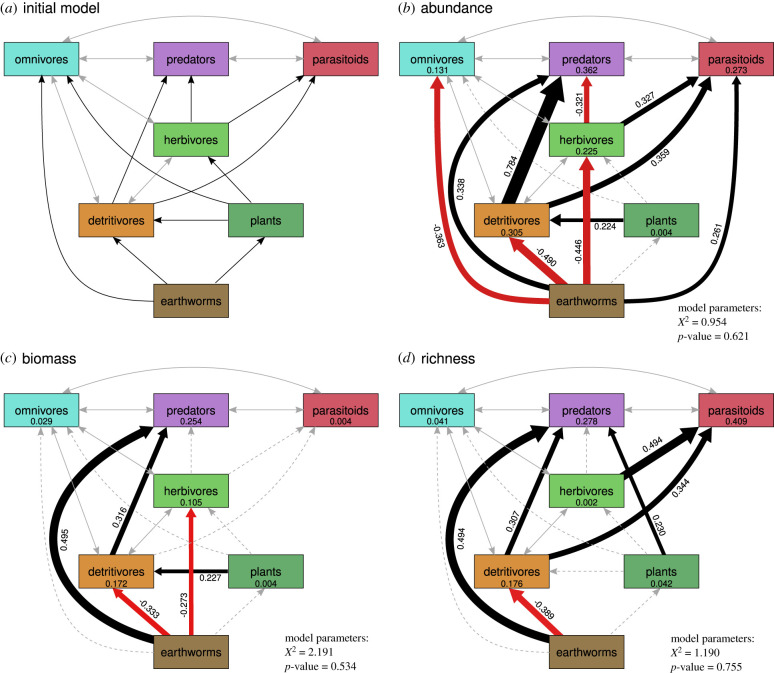
SEMs illustrating direct and indirect effects of earthworm invasion on plants and aboveground arthropod communities. (*a*) Initial model. Final models (*b*–*d*, abundance, biomass, and richness) were obtained following the steps outlined in the electronic supplementary material, SuppInfo §6. Brown boxes represent earthworm biomass. Dark green boxes represent plant total cover (*b*,*c*), or plant species richness (*d*). All other boxes represent trophic-group abundance (*b*), biomass (*c*) or species richness (*d*). Black and red arrows show positive and negative paths, respectively. Grey, double-headed arrows show covariances. Grey dashed arrows show non-significant paths. Numbers next to significant paths are standardized path coefficients. Numbers inside boxes show *R*² values. *N* = 60. For detailed model outputs, see electronic supplementary material, tables S3–S5.

The three SEMs showed direct and indirect effects of invasive earthworms on aboveground arthropod communities (figure 2; electronic supplementary material, tables S3–S5). Earthworm biomass directly increased predator and parasitoid abundance and directly decreased detritivore, herbivore and omnivore abundance (figure 2*b*). It indirectly increased predator abundance via herbivore abundance and indirectly decreased predator and parasitoid abundance via detritivore abundance. Earthworm biomass directly increased predator biomass and directly decreased detritivore and herbivore biomass (figure 2*c*). It indirectly decreased predator biomass via detritivore biomass and parasitoid biomass via herbivore biomass. Earthworm biomass directly increased predator richness and directly decreased detritivore richness (figure 2*d*). It indirectly decreased predator and parasitoid richness via detritivore richness. There were no significant effects of earthworm biomass on plant cover or richness. However, higher plant cover facilitated detritivore abundance and biomass, while plant richness, which was positively correlated to canopy openness (electronic supplementary material, figure S8), facilitated predator richness.

## 4. Discussion

Our observational study highlights belowground invasions as a relevant, yet underrepresented driver of aboveground arthropod decline, with impacts cascading across trophic levels. All feeding types and community properties showed significant responses, with only predator communities directly profiting from earthworm invasion in simple models. Our SEMs illustrate how these net positive effects can be decomposed into direct and indirect effects across trophic levels.

In contrast with our expectations, but in line with some previous work (e.g. [17,23]), earthworms had non-significant negative effects on the plant community. The lack of significance might be caused by earthworms changing plant functional diversity and composition instead of total cover and richness [24,45] or by high variability. Plant cover and species richness supported higher detritivore abundance and biomass, as well as predator richness—presumably by providing more resources and increased habitat heterogeneity [46,47]. Local microclimatic conditions (higher canopy openness) had an additional, indirect effect on aboveground arthropods, via increased plant species richness. This effect was independent of earthworm-invasion effects. Ubiquitous negative effects of earthworm biomass on detritivores, and omnivore abundance, were likely caused by exploitation competition for litter as a resource strongly diminished by earthworm invasion [17,25] and in this forest particularly [35]. Negative effects of earthworm biomass on herbivores might, for example, be caused by earthworm-induced changes in plant secondary metabolites [48], or alternatively via impacts on soil-dwelling herbivore life stages [27,29].

Across community properties, there were consistent and strong, direct positive effects of earthworm biomass on predators, and on parasitoid abundance, that were not mediated by plant richness or cover, or by intermediate trophic levels. Such effects might be mediated by altered habitat structure, such as reduced litter layers [35], or plant community properties [24], but we need further analyses to better understand the underlying mechanisms. It is likely that these seemingly direct effects are mediated by parameters not included in our models. Detritivores facilitated predators and parasitoids, the former as prey, the latter potentially as a host species, or indirectly via cascading positive effects on plants and herbivores (which we did not test; [49]). Herbivores facilitated parasitoids, most prominently in the richness SEM. As herbivore richness was not driven by plant richness, it might respond to plant functional diversity [50], which could also mediate the direct positive effect of earthworms on parasitoids. Finally, the negative relationship between herbivore and predator abundance might indicate that predators have reduced herbivores (top–down effect) instead of herbivores increasing predators (bottom–up effect; [51]).

As one of the first studies reporting effects of invasive earthworms on aboveground arthropod communities (see [28,31]), our paper highlights several topics for future research. First, we need studies investigating the effects of earthworm invasion on vegetation structure, functional diversity and plant metabolites, as well as their impact on arthropod communities [45,48,52]. Furthermore, we need to assess the consequences of belowground invasions and the subsequent aboveground arthropod community changes for consumers of arthropods [12], above–belowground energy flux, ecosystem functions and services [8,53,54]. Future studies should also investigate if earthworm invasion facilitates secondary invasions in aboveground arthropod communities, potentially facilitated by non-native plants [23]. Also, they should assess how earthworm invasion might relate to and interact with other global-change drivers such as climate and land-use change to alter aboveground arthropod communities [55,56]. Finally, given the varying responses of abundance, biomass and richness, our results suggest that including multiple community parameters is key when comprehensively assessing the mechanisms of arthropod community declines under global change.

## Data Availability

R-code, data, and a README file are provided in the electronic supplementary material [44]. The methods section, SuppInfo and README files provide all necessary information about the dataset.
